# Salicylate of Lithia in the Treatment of Rheumatism

**Published:** 1886-11

**Authors:** 


### *SALICYLATE OF LITHIA IN THE TREATMENT OF RHEUMATISM.*

M. Vulpian has observed that the salicylate of soda, although very efficacious in the treatment of acute articular rheumatism and acute attacks of gout, has often no influence upon gonorrhœal rheumatism, and very little upon subacute and chronic articular rheumatism.

On the other hand, in these latter affections, salicylate of lithia appears to have beneficial effect. It acts as a special salt, not only as lithia, or its acid, or its salicylic acid considered alone; the proof of this is that lithia as a carbonate has not produced any good results in the same cases, nor has salicylic acid given as the salicylate of soda. When an acute rheumatism treated by the latter substance has become chronic, it resists its action completely, then good results are obtained by substituting, in the same doses, the salicylate of lithia. Vulpian prescribes it in doses of a drachm and more a day. In larger doses it produces often painful diarrhœa; causes a deafness which does not last.—*Gaz. des hôp.*, Dec. 10, 1885.

